# The association between drugs and repeated treatment with budesonide in patients with microscopic colitis: a retrospective observational study

**DOI:** 10.1177/17562848241240640

**Published:** 2024-03-19

**Authors:** Oliver Bjurström, Pontus Karling

**Affiliations:** Department of Public Health and Clinical Medicine, Umeå University, Umeå, Sweden; Department of Public Health and Clinical Medicine, Umeå University, Umeå, 901 87, Sweden

**Keywords:** acetylsalicylic acid, budesonide, calprotectin, collagenous colitis, lymphocytic colitis, microscopic colitis, non-steroidal anti-inflammatory drugs, proton pump inhibitors, serotonin reuptake inhibitors, statins

## Abstract

**Background:**

Smoking and the use of non-steroidal anti-inflammatory drugs (NSAIDs) acetylsalicylic acid (ASA), proton pump inhibitors (PPIs), serotonin reuptake inhibitors (SSRIs), and statins have been associated with microscopic colitis (MC).

**Objectives:**

We investigated whether these factors were associated with repeated budesonide treatments in patients diagnosed with MC.

**Design:**

Retrospective observational study.

**Methods:**

All patients with a histologically verified diagnosis of MC at our clinic between the years 2006 and 2022 were identified. Baseline factors and drugs prescribed before and after diagnosis were registered. The influence of risk factors on the odds of having a prescription of oral budesonide and the odds of having a second course of budesonide was studied.

**Results:**

Patients with MC (*n* = 183) with a mean age of 62.3 years [standard deviation (SD): 13.3 years] were followed for a median of 5 years (25th–75th percentile 4–10 years) after diagnosis. In all, 138 patients (75%) had at least one prescription of budesonide after diagnosis, and 90 patients (49%) had at least one clinical relapse treated with budesonide. Patients who had been prescribed NSAIDs within 1 year before clinical relapse had higher odds for clinical relapse [odds ratio (OR): 3.70, 95% confidence interval (CI): 1.06–12.9] but there was no increased risk for clinical relapse for the use of ASA (OR: 0.99, 95% CI: 0.39–2.90), PPIs (OR: 1.09, 95% CI: 0.45–2.63), SSRI (OR: 1.41, 95% CI: 0.82–2.44), or statins (OR: 0.83, 95% CI: 0.35–1.99). No association was seen between being a smoker and/or being prescribed NSAID, ASA, PPI, SSRI, and statins at baseline and the odds of having a prescription of oral budesonide within 1 year after diagnosis.

**Conclusion:**

The risk of being prescribed a second course of budesonide is associated with receiving a prescription of NSAIDs but not with the use of ASA, PPIs, SSRIs, and statins.

## Plain language summary

The use of drugs and the odds for patients with microscopic colitis of having a second course of budesonide

Microscopic colitis is a common cause of chronic diarrhea. Previous studies have shown that the use of non-steroidal anti-inflammatory drugs, acetylsalicylic acid, proton-pump inhibitors, serotonin reuptake inhibitors and statins are used more often in patients who later develop microscopic colitis. We aimed to study if these drugs also had an association to an increased risk of disease flares treated with budesonide in 183 patients with known microscopic colitis. Oral budesonide for 6-8 weeks are recommended for disease flares of microscopic colitis. In our study, 90 patients (49%) were prescribed a second course of budesonide probably due to a disease flare. We found a higher odds for being prescribed a second course of budesonide in patients with microscopic colitis who were prescribed non-steroidal anti-inflammatory drugs but no higher risk for the other ‘risk drugs’ for microscopic colitis.

## Introduction

Microscopic colitis (MC) is a common cause of chronic or recurrent non-bloody diarrhea that mainly affects women.^1^ MC is classified into collagenous colitis (CC), lymphocytic colitis (LC), and incomplete MC based on histological examination of colonic biopsies.^2^ The incidence of CC varies from 1.8 to 16.4 per 100,000/year, and for LC from 1.3 to 11.1 per 100,000/year; both are typically diagnosed after the age of 60 years.^3,4^ Budesonide is an effective treatment for MC but approximately half of the patients have clinical relapse after budesonide discontinuation.^5,6^

The pathophysiology of MC is currently not completely understood although smoking^7^ and some drugs^8^ have been associated with an increased risk of being diagnosed with MC. In a large Danish study that used data from the Danish Prescription Register, a strong association was found between current proton pump inhibitor (PPI) use and MC.^9^ Other drugs that have been associated with MC are non-steroidal anti-inflammatory drugs (NSAID), acetylsalicylic acid (ASA), serotonin reuptake inhibitors (SSRIs), and statins.^8^ In recent meta-analyses,^8,10^ all the aforementioned drugs were associated with MC if the control group was randomly selected. However, when the control group was other patients with diarrhea, there was no association between the use of NSAID, SSRIs, or statins, and MC. These studies also demonstrated a lower risk for MC when using PPIs compared to other patients with diarrhea.

It is still uncertain whether smoking and the use of drugs have an impact on the clinical course of patients with MC. In the present study, we investigated the association between the use of NSAIDs, ASA, PPIs, SSRIs, or statins and the risk of being prescribed a second course of budesonide in diagnosed patients with MC.

## Methods

### Study design

This is a retrospective observational study of patients diagnosed with MC in clinical practice. The reporting of this study conforms to the Strengthening the Reporting of Observational Studies in Epidemiology Statement.^11^

### Patients

**Figure 1.**
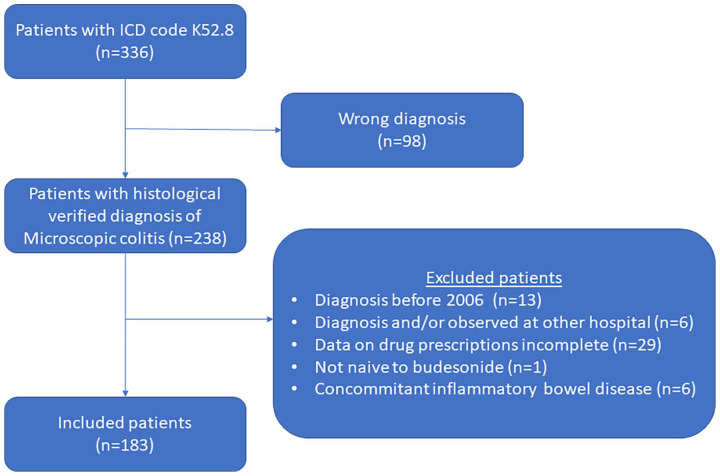
A flow chart that shows the inclusion of patients with MC. MC, microscopic colitis.

All patients within the Norrland University Hospital (Umeå, Sweden) catchment area who were coded with the International Code of Diagnosis (ICD) K52.8 between 1 January 2006 and 31 December 2022 at the Department of Medicine and Department of Surgery were reviewed for inclusion. The inclusion criterion was classified with ICD K52.8 in combination with a histologically proven diagnosis of MC [intraepithelial lymphocytes >20 per 100 surface epithelial cells with (CC) and without (LC) the presence of collagenous subepithelial layer >10 µm].^2^ Exclusion criteria were non-histological-proven diagnosis, diagnosed and/or observed at another hospital, colectomy, concomitant inflammatory bowel disease, and if data regarding drug prescriptions were incomplete (Figure 1).

### Data from the medical chart review and definition of clinical relapse

Baseline data included the date of histologically proven diagnosis of MC, smoking status at baseline (never, former, or current), concomitant diseases at diagnosis (diabetes, coeliac disease, hypothyroidism, and rheumatic disease), and prescriptions of drugs (ASA, NSAIDs, PPIs, SSRIs, and statins) at diagnosis (prescribes within 3 months before diagnosis) and 3 years before diagnosis.

After diagnosis, the yearly prescribed cumulative dose and the number of prescriptions of oral budesonide were calculated for each year. The standard course of budesonide treatment in our clinic is 9 mg daily for 1 month followed by 6 mg daily for 1 month. Patients with MC at our hospital are informed to contact the nurse at the gastroenterology unit in case of clinical relapse. A gastroenterologist then contacts the patient and if he or she judges that the patient has significant symptomatology consistent with MC, a course of budesonide treatment is prescribed.

First, we analyzed baseline risk factors and the odds of having a budesonide course within 1 year of diagnosis. To test the primary aim of our study, that is, how risk factors were associated with a non-favorable disease course, we tested whether these factors were associated with repeated prescriptions of budesonide. In our analysis, we included only patients who had previously been prescribed budesonide and we categorized the patients if they were prescribed a second course of oral budesonide for MC or not. We used a ‘wash-out’ period of 2 months from the date the patient was expected to end the first course of budesonide. We used a ‘wash-out period’ as an attempt to discriminate against patients insufficiently treated for a first flare from a clinical relapse. To determine whether NSAIDs, ASA, PPIs, SSRIs, and statins were associated with a second course of budesonide, we noted if these drugs were prescribed within 1 year before the second budesonide prescription. For those patients who did not have a second course of budesonide, we registered the same drugs prescribed in the last year of observation. Selective cyclooxygenase inhibitors were not included as NSAIDs and serotonin noradrenalin reuptake inhibitors were not included as SSRIs.

### Statistics

IBM-SPSS version 28.0, IBM Corporation, New York, USA was used for statistical calculations. χ^2^ test was used to compare proportions (Fisher’s exact test if small numbers). Ordinal data were compared using the Mann–Whitney test. The Kaplan–Meier curve was used to illustrate the time to clinical relapse and Cox regression analysis was used to test factors associated with time to clinical relapse. Logistic regression was used to test factors for association with clinical relapse. To test for confounders, the logistic regression model was adjusted for age at diagnosis, gender, body mass index (>30 kg/m^2^), smoking status at baseline (current or no current smokers), and the prescriptions of NSAIDs, ASA, PPIs, SSRI, and statins. For the prescription of NSAIDs, a sensitive analysis was performed for the time between the prescription for NSAIDs and a second course of budesonide divided into different categories according to Verhaegh *et al.*^12^ (<90, 90–150, and 151–365 days).

## Results

### Basal characteristics

In all, 183 patients met the inclusion criteria for the study (Figure 1). The mean age of the patients at diagnosis was 62.3 years [standard deviation (SD): 13.3 years] and approximately 79% were women (Table 1). At diagnosis, 18% were current smokers. Table 1 shows the proportion of patients with concomitant diseases and if the patients had been prescribed NSAIDs, ASA, PPIs, SSRI, and statins 3 months or 3 years before diagnosis.

**Table 1.** Baseline characteristics at diagnosis for patients with MC (*n* = 183).

Mean age, years (SD)	62.3 (13.3)
Women, % (*n*)	79.2 (145)
Men, % (*n*)	20.8 (38)
Mean body mass index, kg/m^2^ (SD) (*n* = 160)	25.7 (4.4)
Body mass index >30 kg/m^2^, % (*n*)	15.3 (28)
Smoking at diagnosis (*n* = 149)	
Never, % (*n*)	48.6 (72)
Former, % (*n*)	33.1 (49)
Current, % (*n*)	18.2 (27)
Type of MC	
Collagenous colitis, % (*n*)	40.4 (74)
Lymphocytic colitis, % (*n*)	55.7 (102)
Intermediate MC, % (*n*)	3.8 (7)
Concomitant diseases at diagnosis	
Diabetes, % (*n*)	10.4 (19)
Coeliac disease, % (*n*)	2.2 (4)
Hypothyroidism, % (*n*)	15.8 (29)
Rheumatic disease, % (*n*)	8.7 (16)
Prescribed at least once within 3 years before diagnosis	
NSAIDs, % (*n*)	45.3 (83)
ASA, % (*n*)	21.3 (39)
PPIs, % (*n*)	37.7 (69)
SSRIs, % (*n*)	23.0 (42)
Statins, % (*n*)	26.8 (49)
Prescribed at the time of diagnosis	
NSAIDs, % (*n*)	11.6 (21)
ASA, % (*n*)	14.8 (27)
PPIs, % (*n*)	22.4 (41)
SSRIs, % (*n*)	18.6 (34)
Statins, % (*n*)	21.3 (39)

ASA, acetylsalicylic acid; MC, microscopic colitis; NSAID, non-steroidal anti-inflammatory drug; PPI, proton pump inhibitor; SD, standard deviation; SSRI, serotonin reuptake inhibitor.

### The use of budesonide in the observation period

**Figure 2.**
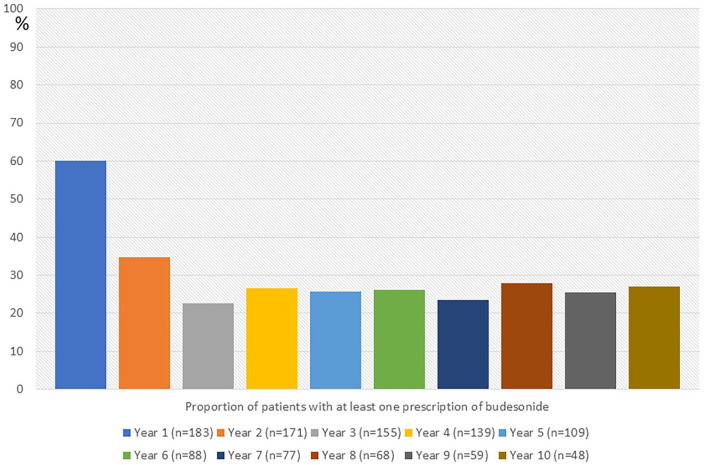
The proportion of patients with microscopic colitis who received at least one prescription of budesonide each year in a 10-year observational period after diagnosis.

The median observation time after diagnosis was 5 years (25th–75th percentiles 4–10 years). In all, 138 patients (75.4%) were prescribed oral budesonide at least once in the observation period. Figure 2 presents the proportion of patients who received at least one prescription for each year after diagnosis. From the third year until the tenth year after diagnosis, the proportion of patients who were prescribed at least one prescription of budesonide for each year remained stable (22–28%). Of the patients who had an observation time of 5 or more years, 30 patients out of 109 (27.5%) were not prescribed budesonide within the first 5 years from diagnosis. Of those patients who had an observation time of 10 years or more, 8 out of 48 (16.7%) were not prescribed budesonide the first 10 years after diagnosis.

### Risk factors at diagnosis and the odds of being prescribed budesonide

In the first year after diagnosis, 110 patients (60%) were prescribed oral budesonide for MC. There was no association between age at diagnosis, gender, obesity, smoking, or being prescribed NSAIDs, ASA, PPIs, SSRIs, or statins at the time of diagnosis or within 3 years before diagnosis and the odds of being prescribed oral budesonide within the first year after diagnosis (Table 2). There were no differences in the proportion of patients with CC and LC who had been prescribed budesonide in the first year after diagnosis (60% *versus* 60%, *p* = 0.96). There was no increase in the odds of being prescribed oral budesonide for the number of risk factors at diagnosis.

**Table 2.** The association between risk factors at diagnosis and the odds of being prescribed budesonide in the first year after diagnosis.

	Budesonide in the first year after diagnosis		Adjusted odds ratio (95% confidence interval)
	Yes (*n* = 110)	No (*n* = 73)	
Age at diagnosis, years (SD)	62.3 (13.7)	62.5	1.00 (0.97–1.03)
Female gender, %	78	81	0.85 (0.40–1.82)
Body mass index >30 kg/m^2^, %	16	14	1.43 (0.59–3.47)
Smoker at diagnosis, %	16	12	1.34 (0.55–3.27)
ASA^a^, %	13	18	0.76 (0.28–2.08)
ASA^b^, %	17	27	0.49 (0.21–1.17)
NSAIDs^a^, %	14	8	2.06 (0.68–6.21)
NSAIDs^b^, %	44	48	0.80 (0.42–1.51)
PPIs^a^, %	24	20	1.10 (0.52–2.33)
PPIs^b^, %	39	36	1.43 (0.73–2.80)
SSRIs^a^, %	19	19	0.90 (0.40–2.01)
SSRIs^b^, %	24	22	1.07 (0.50–2.26)
Statins^a^, %	19	25	0.75 (0.31–1.80)
Statins^b^, %	24	30	0.90 (0.40–2.06)

a Prescribed within 3 months before diagnosis.

b Prescribed within 3 years before diagnosis.

ASA, acetylsalicylic acid; NSAID, non-steroidal anti-inflammatory drug; PPI, proton pump inhibitor; SD, standard deviation; SSRI, serotonin reuptake inhibitor.

### Risk factors for being prescribed a second course of budesonide

**Figure 3.**
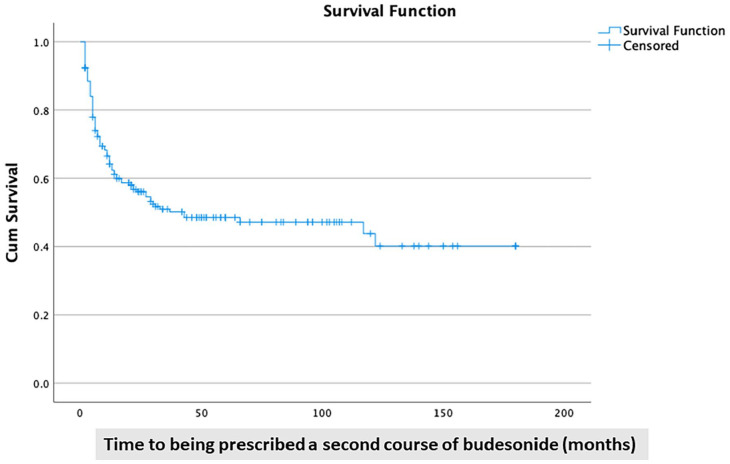
A Kaplan–Meier curve shows the proportion of patients with microscopic colitis who were ‘budesonide-free’ after an initial course of budesonide.

Figure 3 shows a Kaplan–Meier curve with the cumulative proportion of patients who did not receive a second course of budesonide (censored data).

In all, 90 patients (49%) had at least been prescribed two or more courses of budesonide in the observation period. There was no difference in the risk of having a second course of budesonide between patients with CC and LC (47% *versus* 51%; *p* = 0.57). Among the patients who received at least one prescription of budesonide, 90 patients out of 138 (65%) were prescribed a second course of oral budesonide. The median time to the second course of budesonide in those patients was 6 months (25th–75th percentiles 4–14 months). The odds of having a second course of budesonide were higher in patients who had been prescribed NSAID within 1 year before the prescription of the second course of budesonide (Tables 3 and 4). No other drug significantly affected the risk of receiving a second course of budesonide. There was no association between age at diagnosis, gender, obesity or being a smoker at diagnosis, and the risk of being prescribed a second course of budesonide (Tables 3 and 4). Exposure to multiple ‘risk drugs’ did not increase the risk of being prescribed a second course of budesonide.

**Table 3.** The association between risk factors and the odds of being prescribed a second course of budesonide.

	A second course of budesonide		Adjusted odds ratio (95% confidence interval)
	Yes (*n* = 90)	No (*n* = 48)	
Age at diagnosis, years (SD)	61.0 (13.3)	66.1 (12.1)	0.97 (0.93–0.99)
Female gender, %	79	77	1.06 (0.43–2.58)
Body mass index >30 kg/m^2^, %	14	21	0.69 (0.26–1.83)
Smoker at diagnosis, %	18	15	1.06 (0.37–3.1)
ASA^a^, %	16	21	0.99 (0.39–2.90)
NSAIDs^a^, %	21	8	3.70 (1.06–12.9)
PPIs^a^, %	36	31	1.09 (0.45–2.63)
Serotonin reuptake inhibitors^a^, %	21	12	2.07 (0.71–6.04)
Statins^a^, %	30	33	0.83 (0.35–1.99)

a Prescribed within 1 year before the second course of budesonide or the end of observation.

ASA, acetylsalicylic acid; NSAID, non-steroidal anti-inflammatory drug; PPI, proton pump inhibitor; SD, standard deviation.

**Table 4.** A Cox regression model that shows different risk factors associated with being prescribed a second course of budesonide in patients with microscopic colitis (*n* = 138).

	Unadjusted odds ratio (95% confidence interval)	Adjusted odds ratio (95% confidence interval)
Age at diagnosis	0.99 (0.98–1.01)	0.99 (0.97–1.01)
Female gender	1.06 (0.64–1.75)	1.03 (0.62–1.73)
Body mass index >30 kg/m^2^	0.95 (0.52–1.71)	1.03 (0.54–1.93)
Smoker at baseline	0.99 (0.58–1.71)	1.30 (0.73–12.34)
Acetylsalicylic acid^a^	0.93 (0.52–1.65)	1.15 (0.61–2.16)
Non-steroidal anti-inflammatory drugs^a^	1.51 (0.91–2.51)	1.74 (0.95–3.18)
Proton pump inhibitors^a^	1.29 (0.83–1.99)	1.23 (0.75–2.02)
Serotonin reuptake inhibitors^a^	1.27 (0.77–2.12)	1.41 (0.82–2.44)
Statins^a^	1.06 (0.67–1.66)	0.98 (0.59–1.61)

a Prescribed at least once 1 year before the second course of budesonide or the end of observation.

In a sensitive analysis, we found no significant associations between the time the patients were prescribed NSAID (<90, 91–150, or 151–365 days before) and the risk of being prescribed a second course of budesonide (Supplemental Table 1). Among the patients who were prescribed a second course of budesonide, continuous treatment with NSAIDs (1 year or more) was more frequent compared to the patients who did not have a second course of budesonide (12% *versus* 2%; *p* = 0.057).

## Discussion

To our knowledge, the present study is the first to test whether drugs that have been associated with MC increase the odds of being prescribed budesonide. Half of the patients diagnosed with MC in our study had two or more courses of oral budesonide during follow-up. The median time between the first and second course of budesonide was short, that is, 6 months. This is congruent with the findings by Verhaegh *et al.*^13^ who studied the disease activity during the first year after diagnosis in patients with MC and found that 49% of patients had a chronic or relapsing disease during the first year.

In our study, there was no association between being prescribed ASA, PPIs, SSRIs, or statins at diagnosis and being prescribed budesonide in the first year after diagnosis. Furthermore, there was no risk of being prescribed a second course of budesonide using these drugs. In studies that have found an association between MC and the use of PPIs and/or statins, the control group has mainly consisted of subjects from the general population; such associations have not been confirmed in control groups that consisted of patients with other causes of chronic diarrhea or in subjects referred for colonoscopy.^8,14^ The rationale for PPIs and statins as a risk factor for MC are weakly based on current knowledge. First, both PPI^15^ and statins^16^ have shown anti-inflammatory effects on the gastrointestinal tract. Furthermore, statins have been tested as a potential anti-inflammatory drug for inflammatory bowel disease,^17–19^ and in a Swedish population-based study, statins were associated with a lower risk of Crohn’s disease.^20^ Second, statins were shown to indirectly inhibit collagen production by increasing apoptosis of both fibroblasts and myofibroblasts^21^ and increasing the degradation of collagen type I.^22^ Finally, the use of PPIs has been associated with histological resolution in patients with MC.^23^

Among the drugs that had been previously associated with MC, in our study, only prescription of NSAID was associated with the chance of being prescribed a second course of budesonide prescriptions. NSAID is known to affect the mucosa of the gastrointestinal tract.^24^ One-third of rheumatic patients who consume NSAIDs report upper gastrointestinal symptoms and 70% of the patients using NSAIDs have lesions (mucosal erosion, ulceration, subepithelial hemorrhage) on gastroscopy.^25^ These endoscopic lesions are commonly also seen in the small bowel distal to the duodenum.^26^ In addition, patients on NSAID report slightly more symptoms from the lower gastrointestinal tract. For example, diarrhea (6% *versus* 3%), constipation (4% *versus* 2%), and flatulence (4% *versus* 1%) were more commonly reported from patients using NSAIDs compared to those using placebo in randomized controlled trials.^27^ The use of NSAID has not only been associated with MC but also with the risks of being diagnosed with Crohn’s disease and ulcerative colitis.^28^ Finally, the intake of NSAIDs is associated with increased fecal calprotectin levels in patients with a macroscopic normal colonoscopy without inflammatory bowel disease.^29^

This study also presents data on prescriptions for budesonide in the long term and provides indirect evidence of a disease with a relatively favorable course. Although there is a high proportion of patients who are prescribed oral budesonide in the first 2 years after diagnosis, from year 3 and forward approximately three out of four patients have no need for oral budesonide. Inconsistency, in a recently presented abstract (UEG2023:4670) of an extension of the European incidence cohort,^13^ the proportion of patients who were on treatment with budesonide 5 years after diagnosis was 24%. In our study, 27.5% of the patients did not receive any prescription of budesonide during the first 5 years of follow-up. In the mentioned prospective European incidence cohort, only 5% of the patients reported a quiescent course (without the need for treatment). However, this proportion could be underestimated because one out of three patients in that study were lost to follow-up. Furthermore, the patients in that study were probably exposed to a more active follow-up schedule than used in clinical practice which could lead to an increased awareness of the need for treatment. For example, increased monitoring of patients with inflammatory bowel disease is associated with an increased chance to step up treatment.^30,31^ Therefore, we cannot rule out the possibility that the patients with MC in our study have a milder clinical relapse that goes unnoticed in clinical practice. Furthermore, despite a lower need for budesonide treatment in the long term, there are reports that patients with MC show a high degree of residual gastrointestinal symptoms. In a previous study, a high proportion of patients reported residual irritable bowel syndrome-like symptoms^32^ when not on budesonide treatment.

Our study has several limitations. First, we rely only on retrospective data and data depending on documentation in medical records. Second, on the registered prescribed drugs, we do not have data on patient compliance with the drugs and the exact amount of intake of the drugs. Statins, PPIs, ASA, and SSRIs are often prescribed for maintenance therapy over a long period of time but NSAIDs are prescribed mostly sporadically. In Sweden, PPI and NSAID are available as ‘over-the-counter’ drugs, and we only have data on doctor prescriptions for these drugs. However, the tax-financed medical system in Sweden, which includes cost support for prescribed drugs, leads to most long-term PPI users getting their PPIs from doctor prescriptions.^33^ In our study, one can assume that patients with clinical relapse as well as patients without clinical relapse in our study to some extent take NSAIDs bought ‘over the counter’. However, we also assume that a patient who is prescribed NSAIDs uses more of the drug than patients who are not on prescription. Finally, we do not know the exact cause (a side effect of NSAIDs or a flare of MC) for why a patient who is taking NSAIDs receives treatment for clinical relapse of MC.

The strength of our study is that all patients with histologically verified MC in our region are treated at our hospital. In addition, our medical record system allows us to receive data on all doctor prescriptions for the entire Region of Västerbotten.

To conclude, our study shows that patients prescribed ASA, PPIs, SSRIs, and statins do not differ in the odds of being prescribed a second course of budesonide than patients without these drugs, and thus these drugs can be used safely in patients with MC. Patients prescribed NSAIDs also had a higher chance of being prescribed budesonide.

## Supplemental Material

sj-docx-1-tag-10.1177_17562848241240640 – Supplemental material for The association between drugs and repeated treatment with budesonide in patients with microscopic colitis: a retrospective observational study Supplemental material, sj-docx-1-tag-10.1177_17562848241240640 for The association between drugs and repeated treatment with budesonide in patients with microscopic colitis: a retrospective observational study by Oliver Bjurström and Pontus Karling in Therapeutic Advances in Gastroenterology

## Declarations

**Ethics approval and consent to participate:** The study protocol was reviewed by the Swedish Ethical Review Authority (Dnr 2022-060609-01) and judged as a study with no significant ethical considerations. Written informed consent or publication consent from the parent/legal guardian of participants was not required for this retrospective study in accordance with local/national guidelines. The study was performed in accordance with Swedish law for Patient Safety and the directions of the Swedish Data Protection Authority.

**Consent for publication:** The final version of the manuscript was approved by all authors.

**Availability of data and materials:** Data are available from the corresponding author upon reasonable request.
