# Supplementary material for: The association between drugs and repeated treatment with budesonide in patients with microscopic colitis: a retrospective observational study
Source: Ther Adv Gastroenterol. 2024 Mar 19;17:17562848241240640. doi: 10.1177/17562848241240640 (PMC10953108; doi:10.1177/17562848241240640)
Supplement: sj-docx-1-tag-10.1177_17562848241240640 – Supplemental material for The association between drugs and repeated treatment with budesonide in patients with microscopic colitis: a retrospective observational study [file sj-docx-1-tag-10.1177_17562848241240640.docx]

Supplementary Table 1. Crude odds ratio for the chance of being prescribed a second course of budesonide and the exposure to NSAID (based on prescriptions for NSAID).

| Time of last prescription for NSAID before the second course of budesonide | Patients with a second course of budesonide  (n=90) | Patients with no second course of budesonide  (n=48) | Crude odds ratio (95% confidence interval) |
| --- | --- | --- | --- |
| 151-365 days, % | 12 | 2 | 6.8 (0.8-55) |
| 90-150 days, % | 3 | 4 | 0.9 (0.1-5.8) |
| <90 days, % | 6 | 2 | 3.1 (0.3-27.4) |
| Continuous treatment with NSAID^a^, % | 12 | 2 | 4.0 (0.8-52) |

^a^ > one year of valid prescriptions for NSAIDs

reference: Patients with no prescription of NSAID within one year of the end of observation.
